# Single-agent pegylated liposomal doxorubicin (PLD) in the treatment of metastatic breast cancer: results of an Austrian observational trial

**DOI:** 10.1186/1471-2407-11-373

**Published:** 2011-08-24

**Authors:** Michael Fiegl, Brigitte Mlineritsch, Michael Hubalek, Rupert Bartsch, Ursula Pluschnig, Günther G Steger

**Affiliations:** 1 Department of Internal Medicine V/Hematology-Oncoloy, Medical University of Innsbruck, Anichstrasse 35, Innsbruck, Austria; 2 Department of Internal Medicine III, Private Medical University of Salzburg, Müllner Hauptstrasse 48, Salzburg, Austria; 3 Department of Gynecology and Obstetrics, Medical University of Innsbruck, Anichstrasse 35, Innsbruck, Austria; 4 Department of Internal Medicine I/Oncology, Medical University of Vienna, Währinger Gürtel 18-20, Vienna, Austria

## Abstract

**Background:**

In advanced breast cancer, multiple sequential lines of treatments are frequently applied. Pegylated liposomal doxorubicin (PLD) has a favourable toxicity profile and can be used in first or higher lines of therapy. PLD has demonstrated response activity even after prior anthracycline exposure.

**Methods:**

129 consecutive patients with advanced breast cancer, of whom the majority had been massively pretreated, received PLD as monotherapy within licensed approval, for which efficacy and toxicities were documented.

**Results:**

In a routine therapy setting, PLD was administered in a slightly reduced dose (median, 40 mg/m^2 ^per cycle). Response rate (complete and partial remission) was 26%, and stable disease was observed in 19% of patients. Progression-free (PFS) and overall survival (OS) were 5.8 months and 14.2 months, respectively. There was no difference in terms of response and PFS, no matter if patients had already received anthracycline treatment. Interestingly, PFS proved similar regardless whether PLD was administered as palliative therapy in first, second or third line. Furthermore, PFS and OS were similar in patients with response or stable disease, underscoring the view that disease stabilization is associated with a profound clinical benefit. The most common side effects reported were palmar-plantar erythrodysesthesia (17%), exanthema (14%) and mucositis (12%).

**Conclusions:**

Efficacy and toxicity data in these "real life" patients permit the conclusion that PLD is a valuable option in the treatment of advanced breast cancer even in heavily pretreated patients.

## Background

There are numerous systemic therapeutic options available for the tailored treatment of metastatic breast cancer. In addition, therapeutic antibodies and small molecules increasingly complement the therapeutic range of instruments. The choice of systemic therapy follows accepted guidelines (1) but remains largely based on individual factors (2). In case instant response is required, such as rapidly progressive, threatening disease or massive symptoms, combination cytostatic therapy is usually administered; more often, however, monotherapy is the method of choice for patients in whom a long-term stabilization of metastatic disease is the objective. Thus, different therapies can be applied in sequence over a long period of metastatic disease. Although evidence from randomized studies is largely lacking in more advanced disease, administration of multiple treatment lines (e.g., third line and higher) is clinical routine, often drawing out remarkable clinical benefit. However, side effects and cumulative toxicities have to be considered in the choice of a cytostatic to be administered.

Pegylated liposomal doxorubicin (PLD; Caelyx, Aesca, Vienna) is doxorubicin encapsulated in 85 nm vesicles with a lipophilic surface including hydrophilic polyethylene glycol. With this doxorubicin formulation, a prolonged circulation time and differential uptake in abnormally permeable vessels with accumulation in tumor tissue is observed (3). The main advantage of PLD, apart from the clinical benefit provided for breast cancer, is a reduced proportion of patients with classic anthracycline side effects such as cardiotoxicity, hematotoxicity and alopecia (4-10); on the other hand, PLD has been associated with stomatitis and palmar-plantar erythrodysesthesia (PPE), which may prove to be dose-limiting after repeated treatments (10-12).

In an Austrian phase II trial on PLD as second-line monotherapy in metastatic breast cancer, encouraging results with a high rate of clinical benefit at good tolerability were achieved (4). Based on these encouraging results, an observational study was undertaken in Austria to document benefit and toxicities of PLD monotherapy in patients with metastatic breast cancer and cardiac risk factors, who were treated in a routine use setting.

## Methods

### Inclusion criteria and treatment

In accordance with licensed approval and accepted guidelines for the treatment of advanced breast cancer (1), PLD was administered as monotherapy in patients with increased cardiac risk. In this survey on PLD use in Austria, it was prespecified that patients had histologically confirmed, metastatic breast cancer with indication for cytostatic therapy, were older than age 18, and harbored at least one of the following features which might compromise cardiac function: age > 60 years; prior thoracic irradiation (including adjuvant irradiation of the breast); prior anthracycline based therapy; hypertension; and history of cardiac disease, such as infarction and congestive heart failure. Echocardiography results were not documented in this observational study. According to the manufacturer's recommendation, PLD was scheduled in a dose of 50 mg/m^2 ^once every 28 days for 6 cycles. In this survey, aiming at analysing data on PLD use in breast cancer treatment in a "real life" setting, PLD doses and their modification were documented cycle by cycle. The last follow-up data available for this analysis was obtained on 29 September 2010, and the median observation time in this study was 10.1 months.

This was an observational study with treatment of breast cancer by PLD within licensed approval; thus, adhering to the guidelines of the ethics committee of Innsbruck Medical University, formal ethical approval was not required at the time the trial was initiated. Patients were routinely informed about the nature of therapy, but written informed consent did not need to be obtained.

### Response evaluation

Patients who received at least one dose of PLD were assessable for efficacy and safety. Response evaluation was performed after two to four cycles ("mid term" staging) and after completion of therapy, i.e. after 6 cycles as scheduled in the protocol or prior termination in case of progressive disease (PD) or intolerable toxicity. Thus, definitive response was determined at the end of PLD therapy. However, to include temporary therapeutic benefit, an alternative response evaluation was performed, yielding "best response" which was documented separately at both points in time. Response was evaluated according to RECIST criteria for target and non-target lesions, but no central review of radiogramm was conducted. Progression-free survival (PFS) was defined as time from first dose of PLD to disease progression or death, whichever occurred first. Overall survival (OS) was measured from initiation of PLD therapy until final follow-up or death from any cause.

### Statistical considerations

Comparison of response rates between patient subgroups at baseline was carried out using Pearson's chi-square test. Event-related data (PFS and OS) were estimated using the Kaplan-Meier method, and comparison of outcomes between categorical subgroups was made using the log-rank test. Univariate analysis was used to determine which baseline characteristics were associated with PFS and OS. For those found to be associated with PFS and OS (P ≤0.10), hazard ratios were calculated using the multivariate Cox regression analysis. The significance level was set at P < 0.05, in two-sided tests.

## Results

### Patient characteristics

129 patients who had received PLD as monotherapy in 18 Austrian centers were included in this survey between 2003 and 2009. Patient demographics and baseline characteristics including previous therapies and setting of PLD treatment are detailed in Table 1. Two men with metastatic breast cancer were included. All patients were suffering from stage IV breast cancer with different patterns of metastasis. Most patients were characterized by two or more cardiac risk factors; more precisely, 37 patients (29%), 50 (39%), 34 (26%), 4 (3%), and 4 (3%), harbored one, two, three, four or five cardiac risk factors, respectively. Although PLD is licensed to be administered at a dose of 50 mg/m^2 ^per cycle, PLD dose reductions on an individual basis were frequently undertaken. At first cycle, only a minority of patients (n = 46, 36%) received 50 mg/m^2^. Overall, median PLD dose per cycle was 40 mg/m^2^, and only 14 patients (11%) received the scheduled cumulative PLD dose during 6 cycles of 300 mg/m^2^. 17 patients (13%) received more than six cycles (up to 16 cycles) as extended or maintenance therapy (once every month [n = 9] or every second month [n = 8], respectively).

**Table 1:** Patient demographics and baseline charactersitics (n = 129)

Characteristics	N (%)
Median age (range), years	63 (34 - 86)

Median time from diagnosis to PLD (range), years	5.9 (0.1 - 26.7)

Distribution of metastasis	
Bone	84 (65)
Liver	59 (46)
Lymph node	54 (42)
Lung	48 (37)
Skin and sof tissue	42 (33)
Pleura	30 (23)
Abdominal*	16 (12)
Brain	8 (6)
Others+	7 (5)

Metastatic sites involved	
One	19 (15)
Two	54 (42)
Three	26 (20)
Four or more sites	30 (23)

Cardiac risk factors	
Prior anthracycline therapy	77 (60)
Age > 60 years	74 (57)
Hypertension	50 (39)
Prior thoracic irradiation†	39 (30)
History of cardiac disease	35 (27)

Median number of cardiac risk factors (range)	2 (1 - 5)

Previous therapies, setting	
None	4 (3)
Endocrine, adjuvant	66 (51)
Endocrine, metastatic	84 (65)
Endocrine (adjuvant and/or metastatic)	95 (74)
Chemotherapy, adjuvant	77 (60)
Chemotherapy, metastatic	99 (77)
Chemotherapy (adjuvant and/or metastatic)	117 (91)
Anthracycline containing (adjuvant and/or metastatic)	77 (60)
Taxane containing (adjuvant and/or metastatic)	73 (57)
Trastuzumab-containing (adjuvant/metastatic)	14 (11)

PLD line of chemotherapy‡	
First	12 (9)
Second	32 (25)
Third	28 (22)
Fourth and higher	57 (44)

Median number of PLD cycles (range)§	6 (1-6)

Median cumulative PLD dose, mg/m2 (range) §	210 (25 - 300)

*Including patients with peritoneal, ovarian and intestinal metastasis; ^+^pericardial (n = 3), bone marrow (n = 3) and meningeal involvement (n = 1); ^†^including patients with adjuvant irradiation of the breast; ^‡^adjuvant chemotherapy was counted as prior therapy line

^§^PLD cycles within extension therapy (n = 17) not included.

### Response

Response to PLD was evaluated in 120 patients. In the remaining 9 patients, response was not assessable due to premature death (n = 4; fatal congestive heart failure [n = 1], refractory pleural effusion with fatal cardiopulmonal failure [n = 1], end-stage disease and unknown reason in one each), or interruption due to toxicity (n = 4) or lost follow-up (n = 1). Upon termination of scheduled PLD therapy (i.e., after 1 - 6 cycles), an objective response was observed in 34/129 patients (ORR 26%), with CR and PR in 2 (2%) and 32 (25%), respectively (Table 2). ORRs according to patients' characteristics and prior therapies are detailed in Table 2. Of note, 24 patients (19%) who suffered from PD after completion of therapy had PR (n = 2) or SD (n = 22) during PLD therapy; considering this temporary benefit in the "best response analysis", response was observed in 36 (28%) and disease stabilization in 46 patients (36%). Thus, a clinical benefit from PLD, at least a temporary one, was achieved in 82 patients (64%).

**Table 2:** Response to PLD by baseline characteristics

		Response according RECIST after completion of PLD No. of patients (%)						
**Characterstic**	**No. pts**.	**CR**	**PR**	**SD**	**PD**	**NE**	**ORR**	**p**

All patients	129	2 (2)	32 (25)	24 (19)	62 (48)	9 (7)	34 (26)	

Age								
< 63	64	0 (0)	15 (23)	11 (17)	36 (56)	2 (3)	15 (23)	NS
> = 63	64	2 (3)	17 (27)	13 (20)	25 (39)	7 (11)	19 (30)	

Age								
< 50	17	0 (0)	3 (18)	2 (12)	11 (65)	1 (6)	3 (18)	0.382
≥50	112	2 (2)	29 (26)	22 (20)	51 (46)	8 (7)	31 (28)	

No. cardiac risk factors								
1 - 2	88	2 (2)	19 (22)	17 (19)	43 (49)	7 (8)	21 (24)	0.346
≥3	41	0 (0)	13 (32)	7 (17)	19 (46)	2 (5)	13 (32)	

No. metastatic sites								
1-3	99	2 (2)	28 (28)	21 (21)	42 (42)	6 (6)	30 (30)	0.065
4-7	30	0 (0)	4 (13)	3 (10)	20 (67)	6 (9)	4 (13)	

Metastatic site								
Visceral	92	0 (0)	22 (24)	15 (16)	49 (53)	6 (7)	22 (24)	0.321
Nonvisceral	37	2 (5)	10 (27)	9 (24)	13 (35)	3 (8)	12 (32)	

Endocrine therapy								
No	33	2 (6)	4 (12)	7 (21)	6 (49)	4 (8)	6 (18)	0.217
Yes	96	0 (0)	28 (29)	17 (18)	46 (48)	5 (5)	28 (29)	

No. of prior chemos*								
0 - 3	95	2 (2)	28 (30)	22 (23)	38 (40)	5 (5)	30 (32)	0.024
≥4	34	0 (0)	4 (12)	2 (6)	24 (71)	4 (12)	4 (12)	

Prior anthracycline								
No	52	2 (4)	12 (23)	14 (27)	19 (36)	5 (10)	14 (27)	.904
Yes	77	0 (0)	20 (26)	10 (13)	43 (56)	4 (5)	20 (26)	

Prior taxane								
No	56	2 (4)	19 (34)	10 (18)	21 (38)	4 (7)	21 (38)	0.012
Yes	73	0 (0)	13 (18)	14 (19)	41 (56)	5 (7)	13 (18)	

*adjuvant chemotherapy was counted as prior therapy line

In line with a previous investigation (6), ORR upon PLD was independent of prior anthracycline exposure. However, ORR was significantly decreased in patients who had previously received a taxane or both anthracycline and taxane (Table 2).

In the 17 patients with extended PLD therapy (i.e., > 6 cycles), prolonged disease stabilization until termination of PLD was observed in 10 patients, whereas the remaining patients experienced disease progression while on PLD.

### Progression-free survival

**Figure 1:**
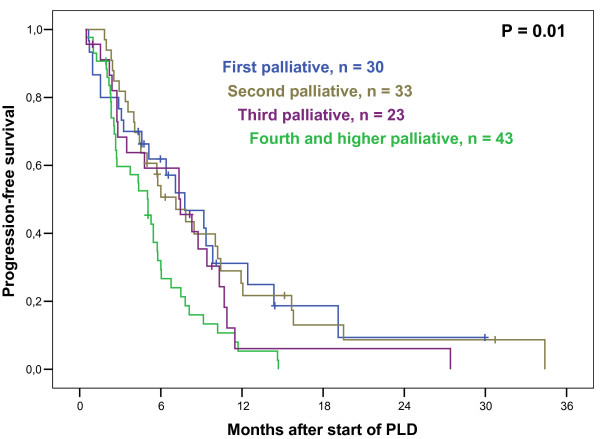
**Progression-Free Survival and line of palliative therapy**. Kaplan-Meier plot illustrating progression-free survival (PFS) in patients with metastasized breast cancer who received liposomal pegylated doxorubicin (PLD) monotherapy. Plotted according to line of palliative treatment in which PLD was applied, median PFS was 7.8 months for patients treated with PLD in first line, 7.1 months in second line, 7.4 months in third line, and 5.0 months in fourth or higher line, which was significantly different when pooled over strata (P = 0.010). Interestingly, pairwise comparisons showed no PFS difference between patients with PLD in first, second or third line. However, PFS was shorter for patients in fourth or higher line of PLD (when compared with first line: P = 0.012; second line: P = 0.004; third line: P = 0.095).

**Figure 2:**
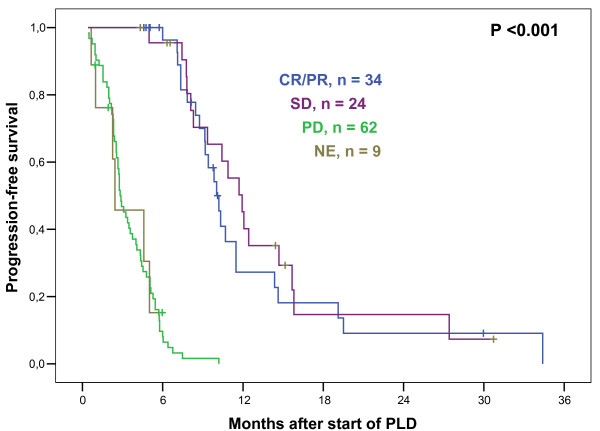
**Progression-Free Survival related to response**. Kaplan-Meier plot illustrating progression-free survival (PFS) in patients with metastasized breast cancer who received liposomal pegylated doxorubicin (PLD) monotherapy. Plotted according to radiographic response after completion of PLD, median PFS was 10.0 months for patients with complete (CR) or partial rememission (PR), 11.9 months with stable disease (SD), 2.8 months with progressive disease (PD), and 2.4 months in which response could not be evaluated (NE), which was significantly different when pooled over strata (P < 0.001). Pairwise comparisons showed no PFS difference between patients with CR/PR and SD.

At last update, 104 patients had documented PD, 4 patients died prematurely without documented PD (see above), and 15 patients were alive without PD at the last follow up. The remaining 6 patients died from breast cancer, but their date of PD was unknown. Thus, for calculation of PFS, these 6 patients were censored at the date of last follow-up without PD. The median PFS for all 129 patients after start of PLD therapy was 5.8 months, well in line with published data (4-10). Interestingly, there was no difference in PFS whether PLD was administered in first, second or third line palliative therapy; however, PFS was significantly shorter when applied in 4^th ^palliative line or beyond (Figure 1). Median PFS according to different clinical characteristics are detailed in Table 3. Prior administration of adjuvant or palliative anthracyclines did not predict for shorter PFS to PLD (Table 3). It was previously reported that disease stabilization on PLD was a positive predictor of survival (8). In line with those data, our results show that patients with SD upon PLD did not differ from CR/PR patients with respect to PFS (Figure 2). A subanalysis was performed in those 48 patients who completed 6 cycles of PLD induction and achieved disease stabilization (SD or better); among those, 17 received further PLD cycles as extended or maintenance therapy (monthly and bi-monthly, respectively; 1-10 cycles, median 3 additional cycles). There was no difference in PFS in patients having received more than 6 cycles compared to those having received 6 cycles of PLD (Table 3).

**Table 3:** Univariate analysis for association of baseline characteristics and therapeutic response with PFS and OS from start of PLD.

Characteristics	Number of patients	Median PFS, months	P value	Median OS, months	P value
All	129	5.8		14.2	

Age					
< 63	64	5.7	0.147	12.5	0.776
≥63	64	7.3		14.6	

Age					
< 50	17	4.0	0.247	16.6	0.880
≥50	112	6.0		14.2	

No. cardiac risk factors					
1 - 2	88	5.3	0.997	14.5	0.163
≥3	41	6.4		11.1	

No. metastatic sites					
1-3	99	7.1	0.021	15.3	< 0.001
4-7	30	3.6		6.0	

Metastatic site					
Visceral	92	5.4	0.034	12.5	0.618
Nonvisceral	37	9.8		14.3	

Prior endocrine therapy					
No	33	5.4	0.305	14.8	0.061
Yes	96	6.0		12.4	

No. of prior chemos^+^					
0 - 3	95	7.3	0.001	14.5	0.01
≥4	34	5.0		10.1	

Prior anthracycline					
No	52	7.8	0.104	15.1	0.057
Yes	77	5.7		10.1	

Prior taxane					
No	56	7.8	0.036	14.3	0.392
Yes	73	5.4		12.5	

Response					
CR/PR	34	10.0	<	17.5	< 0.001
SD	24	11.9	0.001	24.6	
PD	62	2.8		8.6	
NE	9	2.4		3.0	

PLD extension*					
6 cycles, no extension	31	9.8	0.466	21.3	0.516
> 6 cycles	17	10.9		24.2	

*Patients who achieved SD, PR or CR after scheduled PLD (i.e., 6 cycles). ^+^Adjuvant chemotherapy was counted as prior therapy line.

Multivariate Cox regression analysis was performed, and pretreatment baseline characteristics that showed association with PFS in the univariate analysis (Table 3) were included. Independent risk factors for shorter PFS were: greater number (≥4) of metastatic sites (hazard ratio, 1.66; 95% confidence interval [95%CI], 1.05-2.61; P = 0.029); prior taxane-containing therapy (hazard ratio, 1.52; 95%CI, 1.01-2.29; P = 0.044); and greater number (≥4) of prior lines of therapy (hazard ratio, 1.79; 95%CI, 1.15-2.77; P = 0.009).

### Overall survival

At the last follow up, 90 patients had died. Median OS after inititiation of PLD was 14.2 months. OS was significantly shorter in patients with ≥4 metastatic sites and ≥4 prior therapy lines (Table 3). In contrast to PFS, there was a trend towards shorter OS in patients who had had prior anthracycline exposure. In analogy with PFS, patients experiencing disease stabilization had similar OS to those with CR/PR. There was no difference in OS in patients having received > 6 cycles when compared with patients with clinical benefit who discontinued PLD after 6 cycles (Table 3).

In multivariate Cox regression analysis (including the characteristics "number of metastatic sites"; "number of prior chemotherapies"; "prior anthracycline"; and "prior endocrine therapy"), the occurrence of a greater number (≥4) of metastatic sites was the only independent risk factor for shorter OS (hazard ratio, 2.78; 95%CI, 1.75-4.42; P < 0.001).

### Therapies after PLD

Following disease progression after PLD, 79 patients received another line of palliative systemic therapy, whereas 36 patients did not receive further therapy; in the remaining 14 patients, status of subsequent therapy is not known. The most common subsequent therapies were: endocrine therapy (n = 20); single agent chemotherapy (gemcitabine [n = 11]; taxane [11]; capecitabine [8]; platinum derivates [6], PLD reinduction [5]; vinorelbine [3]; non-pegylated liposomal doxorubicin [1]; ralitrexed [1]), combination chemotherapy (CMF [3]); chemotherapy in combination with monoclonal antibodies (docetaxel/trastuzumab [1]; capecitabine/trastuzumab [1]; capecitabine/bevacizumab [1]) or another biologic compound (capecitabine/lapatinib [1]; capacitabine/bortezomib [1]; and monotherapy with a tyrosine kinase inhibitor (lapatinib [1]; gefitinib [1]).

### Safety

The most common side effect reported in this survey was PPE, similar to published evidence, followed by exanthema and mucositis (4, 5, 10, 11). Repeatedly reported side effects are detailed in Table 4. Further adverse events were apoplectic insult, herpes zoster, and panic attack (n = 1 each). We observed that PPE was significantly more prevalent in patients who received > 3 cycles PLD compared to patients receiving only 1-3 cycles (22% vs 8%, P = 0.043). Patients who achieved an objective response or at least SD had PPE more frequently (26% vs 10%, P = 0.016).

**Table 4:** Toxic effects occurring during treatment of advanced breast cancer with PLD (described in at least 2 PLD-treated patients).


**Toxic effects**	**N (%)**

PPE	22 (17)
Grade 1	11 (9)
Grade 2	9 (7)
Grade 3	1 (1)

Exanthema	18 (14)

Mucositis	16 (12)

Nausea/Vomiting	12 (9)

Fatigue	11 (9)

Infectious event	12 (9)

Alopecia (only grade 1 and 2)	7 (5)

Gastrointestinal problems	6 (5)

Respiratory problems	5 (4)

Cardiac problems*	5 (4)

Neutropenia grade 4	3 (2)

Thrombocytopenia grade 4	2 (2)

Thromboembolic event	2 (2)

Cough	2 (2)

Hypersensitivity reaction	2 (2)

*Including 2 patients with severe congestive heart failure, one case of which was fatal. 4 patients with cardiac adverse events during PLD therapy harboured 2 cardiac risk factors, whereas the one patient with fatal exacerbation of heart failure was characterized by 5 cardiac risk factors.

## Discussion

In this observational phase IV study, encouraging results with single-agent PLD in metastasized breast cancer were observed. It is a well known fact that a considerable portion of patients with cancer would not be eligible for inclusion in prospective studies (13), e.g. due to numerous comorbidities and a large number of prior therapies; moreover, data on efficacy of antineoplastic drugs in unselected patients, often compromised by unfavorable performance status, are rare. This study is part of the authors' ongoing efforts to study effects of various treatments in a range of malignancies in a "real life" setting (14-18). Thus, we aimed at defining the role of PLD in 129 consecutive breast cancer patients who were treated within the licensed approval. It must be noted that in an observational study such as ours, patients differ from those in prospective studies. This is especially true with respect to basic characteristics (in the study presented here, there were no exclusion criteria) and in-study procedures. For example, the interval of regular restaging was not strictly pre-specified, a fact which may influence PFS to a certain extent.

We were able to demonstrate that therapeutic results and toxicity rates observed in prospective studies translate nicely into the routine setting (4-11). In the course of data documentation, it was verified that the participating institutions started PLD therapy along the proposed guidelines of administration and had their patients under tight clinical control. Generally, however, we realized that individualization of PLD schedule, predominantly through dose reductions, was frequently carried out, well in line with the observation by other groups (5, 6, 10, 19, 20). In fact, the median dose of PLD administered was 40 mg/m^2 ^per cycle. This dose is less toxic than the licensed 50 mg/m^2 ^per cycle, obviously without compromising therapeutic efficacy (20). Thus, we are convinced that the results presented in our study reflect what is achievable in breast cancer treatment in the routine setting. The data presented here could serve as a benchmark for other surveys on the use of cytostatic monochemotherapy in breast cancer.

We found comparatively low objective response rates and a comparatively high proportion of patients with minor response and disease stabilization, qualifying for SD. Age, prior endocrine or anthracycline therapy did not affect response probability, and response rates were identical between chemo-naive patients and those with one to three prior chemotherapy lines. However, response rate was significantly lower in patients who had had four or more prior therapy lines, a cohort which was relatively large in this survey (26%, Table 2). We observed a higher ORR in the patients who could receive the full course of scheduled six PLD cycles (31/67 patients [49%]), and it became evident that response to PLD can manifest relatively late during therapy. Thus, 9 of the 31 responding patients (29%) had SD in the mid-term staging after two to four cycles.

It appeared that PLD therapy offered favorable survival benefit in the different lines of treatment, comparable up to the third line of palliative therapy, a fact that has not been described yet (Figure 1). This suggests that PLD is a reasonable treatment option even in higher treatment lines when other cytostatic drugs are no longer promising. However, by multivariate analysis, escalating refractoriness also towards PLD, if administered in fourth and higher line, is highlighted; likewise, inferior clinical efficacy by PLD is observed in extraordinarily advanced disease, with 4 or more different metastatic sites involved. Nevertheless, we observed impressive disease stabilizations in a number of patients harboring such unfavourable characteristics, and future work should focus on defining features predicting therapeutic efficacy in such patients.

Patients experienced profound benefit in terms of PFS and OS if they responded to or, more interestingly, had disease stabilization through PLD therapy (Figure 2). This underscores the observation that the achievment of SD by systemic therapy is frequently associated with profound clinical benefit not solely by symptom improvement and gain in quality of life, but also by prolongation of survival (8, 14, 21, 22). In this observational trial, we did not find any indication that extending therapy beyond 6 cycles could be beneficial in terms of PFS or OS. In the GEICAM 2001-01 study, PLD was given as switch maintenance therapy after first line induction by doxorubicin followed by docetaxel. A modest prolongation of time to next therapy but not of OS was observed (23); a general recommendation for PLD use as maintenance therapy cannot be given (24).

In this study, frequencies of toxicities induced by PLD are within the range of expectations (4, 5, 10-12, 19). The typical side effects of PLD therapy, PPE and exanthema (Table 4), appeared to be of minor relevance, probably due to the intensified managment of these toxicities according to the manufacturer's recommendations and published guidelines (25). The lower median dose of PLD may add to this observation (20). The patients in this observational study harbored, to varying extents, cardiac risk. The percentage of patients with cardiac toxicities was small; however, two severe cardiac events (left ventricular dysfunction) occurred during PLD treatment phase, one of which was fatal. Thus, it is emphasized that close monitoring of patients with cardiac risk factors is essential during cytostatic chemotherapy, by frequent clinical examination, and repeated echocardiography and laboratory testing (e.g. NT-proBNP)(26). Grade IV neutropenia and thrombocytopenia occurred in three patients and two patients, respectively; two of them were massively affected by bone marrow carcinosis and, thus, had impaired hematopoietic reserve. Remarkably, partial regeneration of hematopoiesis was achieved after 3 cycles of dose-reduced PLD applications in one of the two patients with grade IV thrombocytopenia.

## Conclusion

The results of this observational study in metastatic breast cancer document well the role of PLD in clinical practice. PLD is a valuable single-agent treatment option in advanced breast cancer, and particularly, for patients with cardiac risk factors and those who had prior therapies including anthracycline.

## Competing interests

MF lectures honoraria Aesca Pharma Austria. Data collection and publication costs were financially supported by Aesca Pharma Austria. The manuscript was prepared independently of Aesca Pharma Austria.

## Authors' contributions

MF, BM, MH, RB, UP, GGS provided patients for the study; MF was responsible for data analysis and manuscript writing; all authors read and approved the final paper.

## Pre-publication history

The pre-publication history for this paper can be accessed here:

http://www.biomedcentral.com/1471-2407/11/373/prepub
